# 

**Published:** 1804-03-01

**Authors:** 


### TO CORRESPONDENTS.

Communications are received from Dr. Moodie, Mr. Marson, Mr. Hardman, and Studiosus.

